# Acknowledgment of Reviewers, 2019

**DOI:** 10.1200/JGO.19.00251

**Published:** 2019-08-27

**Authors:** Ethan Feinstien

Peer review is at the core of scientific progress. It is through the careful critique of peer reviewers that papers are scrutinized, evaluated, and improved. A paper that has been peer reviewed bears the mark of having been inspected and found to be valid and worthy of publication. It is these peer-reviewed papers that ultimately advance our existing understanding of science.

As such, scientific outlets in general, and *Journal of Global Oncology* in particular, owe an immense debt of gratitude to peer reviewers. *JGO* strives to provide the highest quality peer review, so in recognition of Peer Review Week 2019 and its theme of Quality in Peer Review we are pleased to recognize your efforts and thank you for your expertise and time by presenting this list of those who have contributed to our success.

For your hard work in 2019, our heartfelt thank you!

An asterisk indicates reviewers who have completed two or more manuscripts in 2019. A double asterisk indicates reviewers who completed the ASCO Reviewer Mentoring program.

Reem Abdallah

Nafisa Abdel Hafiez

John Tabiri Abebrese

Judith Abrams

Aleesha Adatia*

Bolanle Adegboyega

Pragati Advani*

Shailesh Advani*

David Agom*

Fahad Ahmed*

Oladimeji Akinboro*

Murat Akova

Raafat Alameddine**

Jacqueline Alcalde-Castro

Mazin Aljadiry

Gustavo Almeida

Teresa Almeida Santos

Manwar Alnaqqash

Amin Altijani

Nada Alwan

Chidinma Anakwenze*

Kate Anderson

Rifat Ara

Sanchia Aranda*

Francis Asamoah

Arwa Ashoor

Chite Asirwa

Bassel Atassi

Avani Athauda

Aaron Atkinson

Kirsten Austad

Olutosin Awolude

Burça AydinGovind Babu

Warren Bacorro

Saphalta Baghmar

Hideaki Bando

Pedro Barata*

Ibrhim BariştaUte Bartels

Torben Becker

Asim Belgaumi*

Martine Bellanger

Sara Benitez Majano

Safedin Beqaj

Paulo Bergerot*

Alejandro Berlin

Scott Berry

Nickhill Bhakta

Gouri Shankar Bhattacharyya*

Shristi Bhattarai

Oliver Bogler

Renata Bonadio*

Christopher Booth*

Eric Bouffet

Maria Bourlon**

Sahai Burrowes

Anna Cabanes*

Gabriele Calaminus

German Calderillo

Miguela Caniza

Chrissie Carrington

Carlos Castro

Eduardo Cazap

Pavani Chalasani

Yanin Chavarri

Zhi Cheng

Sung-hee Choi

Supriya Chopra*

Linus Chuang*

Harrison Chuwa*

Carlo Cicero-Oneto

Mishka Cira

C. Norman Coleman*

Michelle Connolly

Olivia Cook

Joanna Coutts

Jennifer Croke

Alberto Jacobo Cunquero Tomás*

Sanju Cyriac

Niloy Datta

Thomas Davis

Shaheenah Dawood

Daphne Day

Henry Ddungu

Roselle De Guzman

Amrita Desai

Haryana Dhillon*

Begoña Díaz de la Noval

Nazli Dizman

Julia Downing*

Pierre-Antoine Dugue

Narjust Duma*

Muhammad Muzzammil Edhi*

Kelechi Eguzo

Uwemedimbuk Ekanem

Brenda Elias

Ahmed Elzawawy*

Josee-Lyne Ethier

Muhammad Mohsin Fareed

Fadi Farhat

Omolara Fatiregun

Andrew Field*

Vaia Florou

Tamer Fouad

Marie Belle Francia*

Sophia Frentzas

Trivadi Ganesan

Gabriel Garcia

Arun Garg

Gail Garvey*

Arunangshu Ghoshal

Meredith Giuliani*

Rodrigo Goncalves

Satsh Gopal

Maja Green

Kalinda Griffiths

Thomas Gross

Surbhi Grover

D Gultekin-Karakas

Bishal Gyawali*

Sabrine Haddad

Khalid Halahleh

Nazik Hammad*

Mhamed Harif

Mutlu Hayran

Valerie Heong

Peter Hesseling

Gwo-Yaw Ho*

Raymond Hohl

Susan Horton

Ali Hosni*

Megan Huchko

Muhammad Husnain

Nzechukwu Ikeri

Chukwuemeka Ikpeazu

André Ilbawi*

Mary Isaacson

Christopher Jackson*

Maheswari Jaganathan

Ishmael Jaiyesimi

Abdul-Rahman Jazieh

Michael Jewett*

Sonali Johnson

Robin Jones

Eric Joo

Josephine Kahiu

Aruna Kamimeni

Christos Karapetis

Ahmed Kaseb

Babita Kataria

Jamal Khader*

Uqba Khan*

Dennis Kim

Peter Kingham

Robert Kridel*

Nicole M. Kuderer*

Vishal Kukreti

Purna Kurkure

Bahar Laderian

Arjun Lakshman

Catherine Lam

Luciana Landeiro

Rita Lawlor

Wenjiao Li

Ditte S. Lind

Jeffrey Lipton

Dorothy Lombe

Adhemar Longatto

Prabhat Malik

Mohandas Mallath

Max Mano

Ben Markman

Deborah Maskens

Charbel Matar**

Aju Mathew*

Amelia McCartney*

Bradley McGregor

Manoj Menon

Monika Metzger

Lauta Mezquita

Vivienne Milch*

Dan Milner

Michael Milosevic

Kamal Mohamed

Mariana Monteiro*

Lesley Moody*

Fabio Moraes

Esther Muinga*

Deborah Mukherji

Esam Murshid

Sreenivasa Murthy

Miriam Mutebi*

Alex Mutombo Baleka

Fumio Nagashima

Mahendra Naidoo

Nataraj Naidu

Govind Nandakumar

Gaurav Narula

Yasodha Natkunam

Twalib Ngoma

Tu Nguyen-Dumont*

Amanda Nogueira

Folakemi Odedina*

Olufunmilayo Olopade

Daniel O’Neil

Sunday Oyerinde

Lydia Pace

Matthew Painschab

Wungki Park

Catriona Parker

Shivani Patel

Catherine Patte

Astrid Pavlovsky

Anh Pham

Leeya Pinder

Richard Powell

Kathy Pritchard-Jones

Ibrahim Qaddoumi

Derek Raghavan*

Lorna Renner

Mark Robson

Gary Rodin

Anya Romanoff

Adrian Sacher

Muhammad Saghir

Suraj Samtani

Flor Sanchez

Hans-Joachim Schmoll

Meltem Sengelen

Yuankai Shi

Lawrence Shulman

Alben Sigamani

Hannah Simonds

Shalini Singh

Bhawna Sirohi*

Jeremy Slone*

Angela Solano

Montserrat Soler

Takayuki Sone

Enrique Soto-Perez-de-Celis*

Melissa Southey

Anthonia Sowunmi

Aaron Sverdlov

Gevorg Tamamyan

Nidale Tarek

Huong Tran

Thuan Tran

Edward Trimble*

Vivien Tsu

Claire Vajdic

Verna Vanderpuye*

Ali Varan

Vinicius Vazquez

Katherine Virgo

Daniel Vorobiof

Horia Vulpe*

Tabassum Wadasadawala

Sara Wahlroos*

Padraig Warde*

Kate Webber

Paul Wissel*

Rebecca Wong

Bilgehan Yalcin*

Suayib Yalcin

Kheng Yeoh

Hyun Jo Youn

Annie Young

Graeme Young

Roberto Zanetti*

Jo Anne Zujewski*

